# Theta Activity in the Left Dorsal Premotor Cortex During Action Re-Evaluation and Motor Reprogramming

**DOI:** 10.3389/fnhum.2018.00364

**Published:** 2018-09-21

**Authors:** Giovanni Pellegrino, Leo Tomasevic, Damian Marc Herz, Kit Melissa Larsen, Hartwig Roman Siebner

**Affiliations:** ^1^Danish Research Centre for Magnetic Resonance (DRCMR), Centre for Functional and Diagnostic Imaging and Research, Copenhagen University Hospital Hvidovre, Hvidovre, Denmark; ^2^San Camillo Hospital IRCCS, Venice, Italy; ^3^Department of Neurology, Copenhagen University Hospital Bispebjerg, Copenhagen, Denmark; ^4^Queensland Brain Institute, The University of Queensland, Brisbane, QLD, Australia

**Keywords:** action selection, motor reprogramming, theta, EEG, dorsal premotor cortex, motor, performance, reaction time

## Abstract

The ability to rapidly adjust our actions to changes in the environment is a key function of human motor control. Previous work implicated the dorsal premotor cortex (dPMC) in the up-dating of action plans based on environmental cues. Here we used electroencephalography (EEG) to identify neural signatures of up-dating cue-action relationships in the dPMC and connected frontoparietal areas. Ten healthy subjects performed a pre-cued alternate choice task. Simple geometric shapes cued button presses with the right or left index finger. The shapes of the pre-cue and go-cue differed in two third of trials. In these incongruent trials, the go-cue prompted a re-evaluation of the pre-cued action plan, slowing response time relative to trials with identical cues. This re-evaluation selectively increased theta band activity without modifying activity in alpha and beta band. Source-based analysis revealed a widespread theta increase in dorsal and mesial frontoparietal areas, including dPMC, supplementary motor area (SMA), primary motor and posterior parietal cortices (PPC). Theta activity scaled positively with response slowing and increased more strongly when the pre-cue was invalid and required subjects to select the alternate response. Together, the results indicate that theta activity in dPMC and connected frontoparietal areas is involved in the re-adjustment of cue-induced action tendencies.

**Highlights**

- Incongruent go-cues slow down response time in a pre-cued alternate-choice task.
- Response slowing results from the need to re-evaluate the cue and its associated action.
- Re-evaluation is characterized by a selective increase in frontoparietal theta band activity.
- The dynamics of theta activity in left dPMC scales with response slowing.

## Introduction

In everyday life, we constantly adjust our actions according to external cues, enabling flexible adjustments to changes in the environment. A sudden change in the environment may prompt us to reconsider our action plans. This re-evaluation may lead to a confirmation of the planned action or to the selection of an alternative action. A bilateral dorsal and mesial frontoparietal network has been implicated in the re-evaluation of pre-planned actions based on external cues, including premotor cortex (PMC), posterior parietal cortex (PPC), supplementary motor area (SMA) and medial prefrontal cortex (Paus, 2001; Monsell, 2003; Nachev et al., 2008; Mars et al., 2009; Buch et al., 2010; Neubert et al., 2010; O’Doherty, 2011; Hartwigsen et al., 2012; Mutha et al., 2014). The dorsal PMC (dPMC) plays a key role in the updating of pre-planned actions and non-routine stimulus-response mapping (Ward et al., 2010; Hartwigsen et al., 2012; Moisa et al., 2012; Hartwigsen and Siebner, 2015). The role of the left dPMC (L dPMC) in cue-based adjustments of action selection has also been studied invasively in behaving animals, exploring preparatory activity of small neuronal populations during trained sensorimotor tasks (Mushiake et al., 1991; Cisek and Kalaska, 2005). The dPMC is also relevant to functional recovery in patients with motor paresis after stroke (Seitz et al., 1998; Johansen-Berg et al., 2002; Fridman et al., 2004; Di Pino et al., 2014). In humans, the involvement of dPMC in re-evaluation of existing action plans has been studied with functional brain mapping techniques, such as functional magnetic resonance imaging (fMRI; Picard and Strick, 2001; Rae et al., 2014) or electroencephalography (EEG; Gratton et al., 1990; Eimer et al., 1995; Verleger et al., 2009). Others have used focal transcranial magnetic stimulation (TMS) to perturb neural processing, trace changes in cortical excitability, or interface TMS with fMRI (Koch et al., 2006; O’Shea et al., 2007; Kroeger et al., 2010; Ward et al., 2010; Duque et al., 2012; Hartwigsen et al., 2012; Bestmann and Duque, 2015).

Although it is widely accepted that brain oscillations make critical contributions to action selection and control (Humphries et al., 2006; Tombini et al., 2009; Deiber et al., 2012; Cheyne, 2013; Brittain and Brown, 2014), it remains to be clarified which oscillatory activity patterns emerge in the dPMC and connected frontoparietal brain regions during cue-induced re-evaluation of pre-planned actions.

In this EEG study, we recorded cortical oscillatory activity during a novel pre-cued alternate-choice task. Simple geometric shapes cued button presses with the right or left index finger. The shapes of the pre-cue and go-cue differed in two third of trials. Partially incongruent go-cues required a re-evaluation of the motor plan, but no change of the previously planned motor output (motor re-evaluation). Incongruent go-cues required a re-evaluation of the go-cue and its associated action (motor reprogramming). We hypothesized that those trials requiring a re-evaluation of cue-action associations would show an increase in oscillatory activity during the response period.

We expected that the increase in activity would be most prominent in the dPMC and interconnected frontoparietal areas and would positively correlate with the slowing in response time.

Although action re-evaluation and motor reprogramming also depend on the activity and cortical regions localized deep near the longitudinal fissure, such as anterior cingulate cortex (ACC), and subcortical structures such as the subtalamic nucleus (STN; Wessel and Aron, 2017), we only focused on the cortical surface to which EEG is sensitive and accurate.

## Materials and Methods

### Participants and Study Design

**Figure 1:**
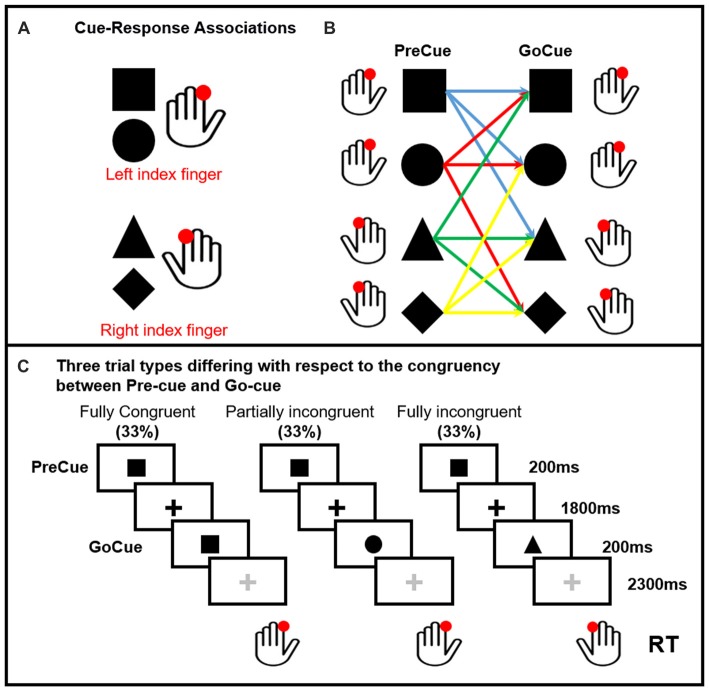
Pre-cued alternate-choice task. Participants were asked to respond as fast as possible to a GoCue with a correct button press. Arbitrary shape-response associations were learned by the participants prior to the experiment. Panel **(A)** Cue-response associations: the square and circle required a button press with the left index finger. The triangle and diamond required a button press with the right index finger. The cue-response associations were varied across individuals, but kept constant within subjects. Panel **(B)** shows all the possible PreCue GoCue combinations. Panel **(C)** Trial structure: a PreCue appeared for 200 ms on the screen and predicted the movement with a probability of 66%. A GoCue appeared 1,800 ms after the PreCue had disappeared and was presented for 200 ms. In one third of the trials, PreCue and GoCue were identical (i.e., fully congruent trials). Hence, the PreCue predicted the GoCue and the action. In 33% of trials the shape of the GoCue differed from the shape of the PrCue, but the GoCue instructed the same movement as the PreCue (i.e., partially incongruent trials). Hence, the altered go-cue required only a re-evaluation of the cue but no change in selection of the pre-cued response. In the last third of the trials, the PreCue and GoCue differed in shape and coded different actions (i.e., fully incongruent trials). In this trial condition, not only the go-cue needed to be re-evaluated, but also the pre-cued response needed to be suppressed and the alternate response had to be selected (i.e., motor reprogramming).

Ten healthy volunteers participated in the study. All participants but one were right handed (Oldfield, 1971). None was under medications or had history of neurological or psychiatric disorders. The protocol was approved by the Ethics Committee of the Capital Region of Denmark. All subjects gave written informed consent in accordance with the Declaration of Helsinki. Subjects performed a visually precued, bimanual two-choice reaction time (RT) task (Figure 1). Two shapes (square and circle) were associated to the movement of the left finger, a triangle and a diamond to the movement of the right finger. One of these four cues were randomly presented on the screen (PreCue). Each shape of the PreCue could be followed (GoCue) by the same shape (*Fully Congruent*, 33%), a different shape triggering the movement of the same side (*Partially Incongruent*, 33%) and a different shape triggering the movement of the opposite side (*Fully Incongruent*, 33%). Hence, in 66% of the trials the PreCue validly predicted the movement side (*Fully Congruent* + *Partially Incongruent*). In further details, this task allowed to assess the cortical mechanisms related to motor re-evaluation (*Partially Incongruent* vs. *Fully Congruent*) and motor reprogramming (*Fully Incongruent*).

The task consisted of five runs of 120 trials each (600 trials in total, 200 per condition). The PreCue and GoCue appeared for 200 ms each. The PreCue-GoCue inter-stimulus interval was 2 s, while the GoCue-PreCue interval was 2.5 s. A fixation cross was presented all along the experiment. The color of the fixation cross was black in the PreCue-GoCue interval and gray in the GoCue-PreCue period. The shapes and the fixation cross had all the same size. The subjects were instructed to perform a button press with their left or right index finger (“A” and “L” buttons of a computer keyboard respectively) as quickly and accurately as possible. Stimulus presentation and response recordings were obtained using Psychopy software (Peirce, 2007, 2008). EEG data were acquired all along the experiment. As the overall goal of the study was to investigate the mechanisms linked to motor reprogramming, the analysis was focused on data aligned to the GoCue.

### EEG Recording and Analysis

EEG was acquired from 64 electrodes mounted on an elastic cap (Easycap). Data were sampled at 5,000 Hz, amplified using a BrainAmp MRI plus system and recorded using the BrainVision Recorder (Brain Products GmbH, Germany). Impedances of all electrodes were kept below 5 kΩ. Pre-processing was performed using EEGLAB (Delorme and Makeig, 2004) and in-house code (MATLAB R2014b, The Mathworks, Natick, MA, USA) and included the following steps: (a) downsampling to 1,000 Hz; (b) band-pass filter (0.3–200 Hz); (c) notch filter (49–51 Hz); (d) visual inspection to remove bad channels; (e) average re-referencing; (f) data epoching (epochs of 2.45 s, from −1.7 s to 0.75 s after the GoCue); (g) visual rejection of bad epochs; (h) independent component analysis (ICA) to remove artifacts, such as eye movements, eye blinks, muscle artifact; and (i) interpolation of bad channels using spherical splines. Preliminary analysis aimed at identifying the frequency band relevant to the task focusing on the time interval from 0 ms to 500 ms after the GoCue and on theta (3–6 Hz), alpha (8–13 Hz), beta (15–30 Hz) and gamma (30–60 Hz) bands. The time-frequency (TF) decomposition was performed at sensor level using FFT and Hanning window tapering (500 ms with 90% overlap), setting time-steps of 100 ms. To avoid border effects and achieve a robust estimation of the low frequency activity, frequency decomposition was applied on a wider time-window, spanning between −1,700 ms and 750 ms, relative to onset of the GoCue. The activity of interest (0–500 ms) was normalized considering a PreCue baseline (−1,000 to 0 ms). For the frequency bands significantly different across conditions a source imaging analysis was performed using Brainstorm (Tadel et al., 2011). This assessment aimed at identifying the cortical regions relevant to motor reprogramming, the relationship between brain activity originating in such regions and RT and the time dynamic of the generation of brain oscillations. The head model for the source imaging was built from an anatomical template (Colin27). Cortical surface was reconstructed via Freesurfer and tessellated into 8,000 vertices (Dale et al., 1999). The forward model was computed applying the OpenMEEG Boundary Element Method (BEM; Gramfort et al., 2010), with three layers and a conductivity of 0.33 S/m for the brain, 0.165 S/m for the skull and 0.33 S/m for the skin (brain-to-skull ratio: 1/20; Pellegrino et al., 2016a,b, 2018; von Ellenrieder et al., 2016; Hedrich et al., 2017). The inverse problem was solved by using a whitened and depth-weighted linear L2-minimum norm estimate, with the dipole orientations constrained to be normal to the cortex. A common imaging Kernel was applied to compute single trial cortical reconstructions. For each trial and each cortical vertex, the TF decomposition was estimated from 0 ms to 500 ms and from 3 Hz to 6 Hz (this was the only frequency band significantly different across conditions at the preliminary sensor-level analysis, see “Results” section). The following regions of interest (ROIs) were manually identified on the cortical surface according to previous literature: Bilateral Medial Prefrontal Cortex (MPFC; motor part; Paus, 2001; Ramnani and Owen, 2004), Left and Right Dorsolateral PreFrontal (DLPF) Cortex (Simons and Spiers, 2003), Bilateral Rostral SMA, Bilateral Ventral SMA (Paus, 2001; Nachev et al., 2008), Left and Right primary motor cortex (M1; Yousry et al., 1997), Left and Right dorsal and ventral PMC (Mayka et al., 2006), Left and Right Posterior Parietal, Left and Right inferior frontal gyrus (IFG; Petrides and Pandya, 2002). All these regions are well-known for being engaged in motor reprogramming and are compatible with the topographical distribution of the effect found on sensor level (see “Results” section). The TF of all vertices belonging to each ROIs was then averaged. As compared to applying TF decomposition to the average time-course of the ROIs, this procedure is more robust and potentially less prone to bias related to EEG cancellation phenomena due to cortical folding (Chowdhury et al., 2018). Although ACC and the STN are two key players in action re-evaluation and motor reprogramming, as well as in conflict solving (Botvinick et al., 2004; Sohn et al., 2007), we did not place a seed in these regions as the accuracy of distributed magnetic source imaging in estimating deep sources is very low and questionable (Koessler et al., 2015; Pellegrino et al., 2018).

### Statistics

Behavioral data were analyzed using IBM SPSS Statistics v22. Analysis of RT was carried out with a Repeated Measure ANOVA. The rate of errors was compared across conditions using a Chi-Square test. For the preliminary analysis on sensor level, the frequency content of the three Conditions (*Fully Congruent*, *Partially Incongruent*, *Fully Incongruent*) were compared using the paired parametric approach embedded in EEGLab. The effect of brain oscillations on motor performance was assessed using linear regressions performed with IBM SPSS Statistics v22 on data extracted from Brainstorm source imaging, as further explained in the “Results” section. The significant level was set to *p* < 0.05.

## Results

### Motor Performance

We first present the analysis of the motor performance with a focus on two measures: RT and error rate.

**Figure 2:**
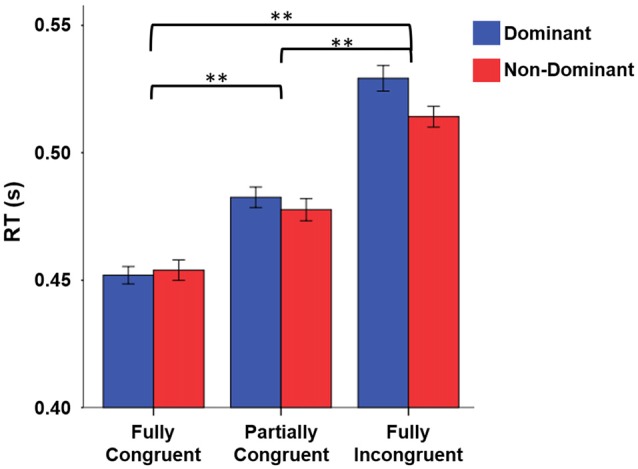
Reaction time (RT) differences by Condition by Side. RT was significantly higher for Fully Incongruent than Partially Congruent than Fully Congruent. No differences were found between Dominant and Non-Dominant side. **p < 0.001.

When looking at RT, we found that it strongly depended on task difficulty (Figure 2). The repeated measure ANOVA with the factors *Condition* (*Fully Congruent*, *Partially Congruent*, *Fully Incongruent*) and *Side* (*Dominant* and *Non-dominant*) demonstrated a *RT* differences across conditions (*F*_(2,18)_ = 48.840, *p* < 0.001) but no significant difference between sides (Factor *Side* and *Side* by *Condition* interaction: *p* > 0.200 consistently). The *post hoc* analysis confirmed that the RT was higher for *Fully Incongruent* vs. *Partially Incongruent* (*p* = 0.001) and *Partially Incongruent* vs. *Fully Congruent* (*p* < 0.001).

A similar pattern emerged for error rate. *Fully Incongruent* had more errors than *Partially Incongruent* and the latter more than *Fully Congruent* (Chi-Square 84.763, *p* < 0.001; *Fully Incongruent* 137 (6.85%), *Partially Incongruent* 60 (3.00%), *Fully Congruent* 36 (1.80%)). Similarly to RT, also in this case no significant difference was found between *Sides* (*p* > 0.05). The cumulative number of errors as well as the number of missed responses were overall quite low (224 (3.73%) and 37 (0.6%), respectively).

In summary, both measures confirmed that there was an increasing task difficulty, with *Fully Incongruent* being more challenging than *Partially Incongruent* and the latter more challenging than *Fully Congruent*. On the basis of these behavioral results, we focused our EEG analysis on the correct trials only and pooled together data of dominant and non-dominant hand.

### EEG Analysis

**Figure 3:**
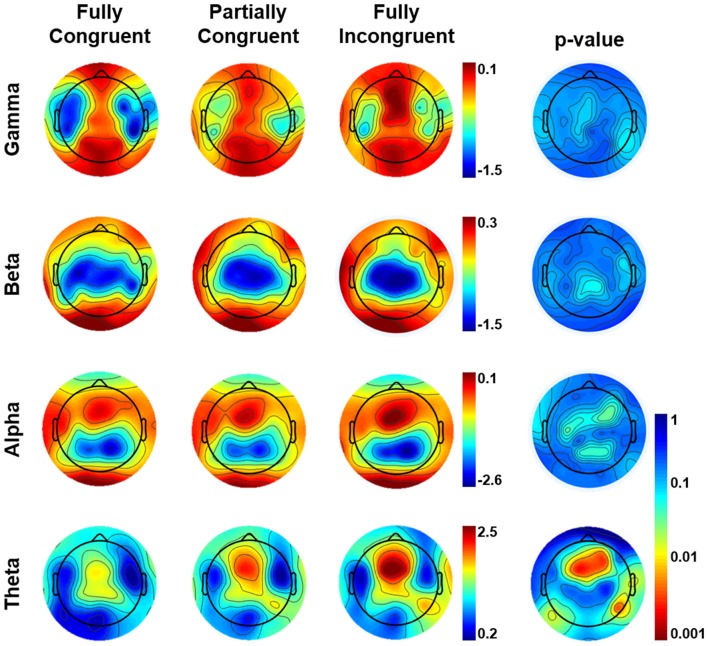
Topographical distribution of frequency power by Condition by frequency band in the (0–500) ms time-window of interest. The most-right column shows the Bonferroni corrected *p*-value of the comparison across conditions. From Fully Incongruent to Partially Congruent to Fully Congruent there is an increasing amount of theta activity. To be noted that no significant differences were found for other frequencies.

The results of the sensor-level analysis are reported in Figure 3. They show that theta activity followed a similar pattern as motor performance. In details, *Fully Incongruent* displayed a significant higher theta than *Partially Incongruent*, and the latter had significantly more theta than *Fully Congruent*. Notably, this feature was very specific for theta, as none of the other frequency bands disclosed any significant difference across conditions.

**Figure 4:**
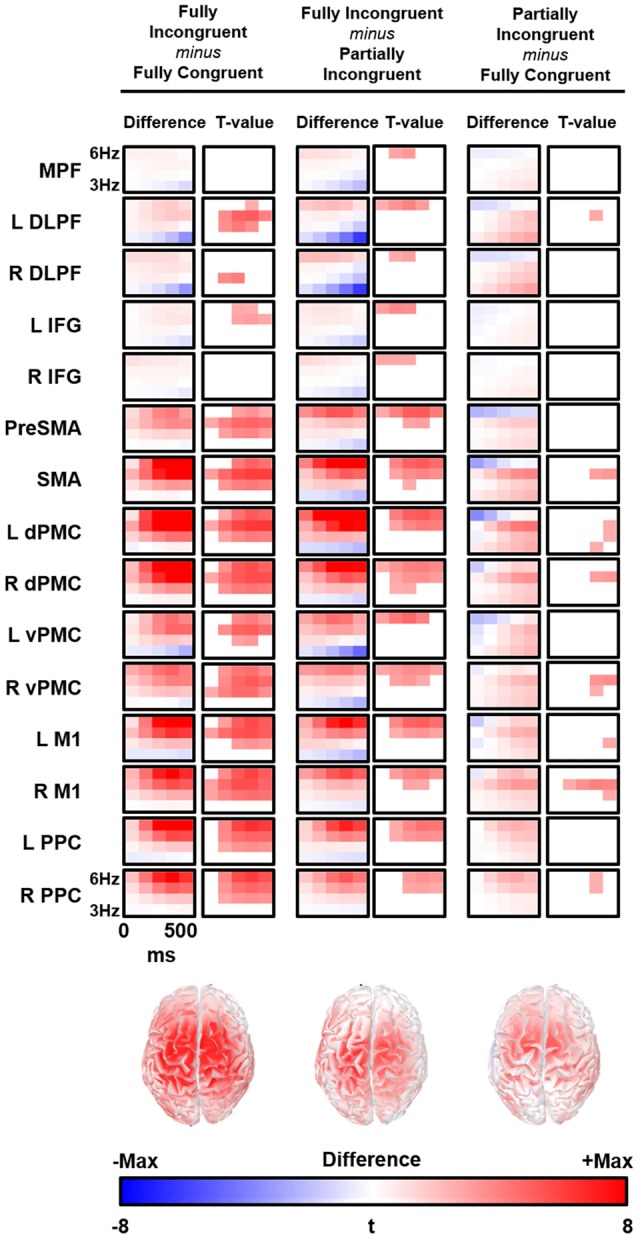
Time-frequency (TF) difference (left column) and the corresponding t-map (right column) is shown for each regions of interest (ROIs). The maps of the *t*-value are thresholded at Bonferroni corrected *p* < 0.05. Bottom line shows the cortical distribution of theta activity difference between conditions at 300 ms and 5 Hz. R, Right; L, Left; MPFC, Bilateral Medial PreFrontal Cortex; DLPF, Dorsolateral PreFrontal; IFG, Inferior Frontal Gyrus; PreSMA, Bilteral Pre Supplementary Motor Area; SMA, Bilateral Supplementary Motor Area; dPMC, Dorsal PreMotor Cortex; vPMC, Ventral PreMotor Cortex; M1, Primary Motor Cortex; PPC, Posterior Parietal Cortex.

We followed-up on these findings with a source analysis restricted to the theta band. The main results are reported in Figure 4. The evaluation of theta temporal dynamic revealed that theta differences between conditions began before the movement onset (average *RT* from 450 ms to 520 ms for *Fully Congruent* and *Fully Incongruent*, respectively). Motor reprogramming (*Fully Incongruent* vs. *Fully Congruent)* was characterized by higher theta in multiple regions, and especially in dPMC, supplementary motor cortex and PPC. Conversely, motor re-evaluation (*Partially Incongruent* vs. *Fully Congruent)* was characterized by higher dPMC and supplementary motor theta activity.

### Effect of Theta Activity on RT

As we had found that more challenging conditions (motor re-evaluation and motor reprogramming) had longer response times and higher theta activity arising from multiple cortical regions, we were interested to find whether this relationship also explained inter-individual variability in RT within a given experimental condition. Therefore, we performed condition-specific analysis to test for a link between differences in motor performance and differences in theta activity for each experimental condition and explored in which cortical areas the condition-specific expression of theta activity scaled with motor performance.

**Figure 5:**
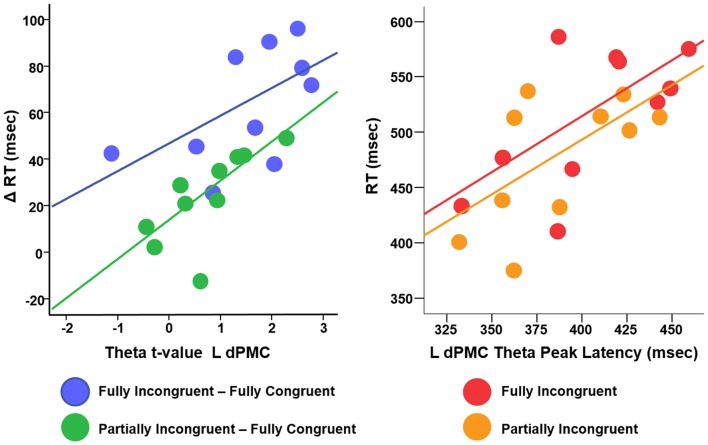
Relationship between difference RT cost (ΔRT) and theta *t*-values (left panel) and between RT and theta peak latency (right panel).

We conducted a multiple linear regression analysis with *RT* differences between conditions as dependent variable and two predictors: the three experimental conditions *CondDiff* and *t*-scores of the theta of the ROIs. *CondDiff* had three levels: (a) *Partially Incongruent minus Fully Congruent*; (b) Fully Incongruent *minus Partially Incongruent*; and (c) *Fully Incongruent minus Fully Congruent*. Whereas *CondDiff* was pushed into the model, *t*-values of all ROIs were added in a stepwise fashion. *CondDiff* alone explained about 40% of *RT* variance (*F* = 10.414, *p* < 0.001). L dPMC was the only region improving significantly the model (about 60% of variance explained; *F* = 14.952, *p* < 0.001; Standardized beta = 0.468, *t* = 3.742, *p* = 0.001), thus suggesting that differences in motor performance scale with difference in L dPMC theta. Therefore, this model provided strong evidence that L dPMC, which was the target region of our task and experimental design, was a main driver of motor performance. To be noted, no other regions gave a significant additional contribution to explain differences in RT. In more details, the positive beta coefficient denoted that the higher was the difference in theta, the higher was the difference in performance between conditions (Figure 5). We also fitted an additional model with an interaction term, but no significant differences were found. This additional finding revealed that the relationship between L dPMC and RT was similar across *CondDiff* levels.

In summary, this analysis demonstrated that, although motor re-evaluation and motor-reprogramming are characterized by differences in theta band arising from dPMC and other interconnected regions, only theta activity in L dPMC was linearly related to motor performance. Therefore, the following analyses focus on L dPMC theta activity and further investigates how it relates to motor performance.

While we previously showed that differences in theta activity relate to differences in task performance, here we investigated whether the amount of theta had an impact on the motor performance within our three experimental conditions (*Fully Congruent*, *Partially Incongruent*, *Fully Incongruent*).

A linear regression with *RT* as dependent variable, *Condition* and L dPMC theta as predictors showed a negative association between *RT* and amount of theta oscillations (*F* = 4.090, *p* = 0.017; Standardized beta = −0.370, *t* = −2.29, *p* = 0.03), similar for the three conditions (interaction term *p* > 0.200). This analysis demonstrated that faster responses significantly depend on higher theta and that this relationship was similar for *Fully Congruent*; *Partially Incongruent and Fully Incongruent*.

We finally investigated the effect of theta temporal dynamic on motor performance. We defined as *Peak Latency* the average time (per *Condition* per *Subject*) when theta reached its peak in amplitude. Although the resolution of TF decomposition was set to 100 ms, Peak Latency could assume any value in the (0–500 ms) time window, being an average over many trials. We computed a linear regression with *RT* as dependent variable, *Condition* (three levels: *Fully Congruent*; *Partially Incongruent*; *Fully Incongruent*) and *Peak Latency* as predictors. This analysis revealed a significant and positive relationship between *Peak Latency* and *RT* (*F* = 8.23, *p* = 0.001, Adjusted *R*^2^ = 43%, Standardized beta = 0.569, *t* = 3.93, *p* = 0.001), suggesting that an earlier theta build-up scales with faster responses. Similarly to the previous analysis, also in this case the relationship did not significantly change across *Conditions*, as the interaction term was not significant (*p* > 0.200; Figure 5). In other words, for all the three conditions, earlier the theta in L dPMC reached its peak in L dPMC, faster was the response.

## Discussion

In this study, using a novel pre-cued RT task, we were able to demonstrate that theta activity generated in the L dPMC is a neural signature of motor re-evaluation and motor re-programming. This finding is in good agreement with the notion of a dorsomedial visuomotor “action pathway,” as recently discussed by Gallivan and Goodale (2018).

With our novel task we wanted to disentangle the effect of motor reprogramming and motor re-evaluation. Action selection was therefore decomposed in three different components: a baseline consisting in releasing a previously prepared motor action (*Fully Congruent*), a motor re-evaluation where the subject had to reassure that the movement previously prepared could be released (*Partially Incongruent*) and an entire motor reprogramming when a cue signaled to execute a different movement (*Fully Incongruent*; Figure 1). We demonstrated that both motor re-evaluation and motor reprogramming have a behavioral cost (longer RT) and, more importantly, that this cost pairs differences in theta activity in L dPMC. Theta increase in motor re-evaluation and motor-reprogramming fully scaled with the RT costs.

Theta activity over the fronto-central cortical regions has been traditionally called midline theta and seemed to be largely generated in the medial prefrontal cortex (Ishii et al., 1999). It often occurs in conjunction with cortical synchronization in other frequency bands (Mizuki et al., 1982; Tombini et al., 2009) and it has been often interpreted as a diffuse and non-specific activation. It increases in a number of tasks with a relevant cognitive load (Nigbur et al., 2011), ranging from internalized attention and positive emotional states (Aftanas and Golocheikine, 2001), to learning (Laukka et al., 1995), memory (Jensen and Tesche, 2002; Tombini et al., 2012; Hsieh and Ranganath, 2014), stimulus response conflicts (Luu et al., 2004; Nigbur et al., 2012) and spatial conflict processing (Cohen and Ridderinkhof, 2013). In the context of online action control, theta activity is expressed when overlearned sensorimotor response mapping is challenged by external cues and preferentially expressed in the dPMC. The relationship of this activity pattern to neural activity more generically implicated in conflict solving needs to be addressed in future studies that involve additional tasks which do not require a motor response.

Our study, however, revealed that, in the field of motor control, theta activity is both task and spatially specific. First, although motor control depends upon the modulation of oscillations at multiple frequencies (Neuper et al., 2009; Tombini et al., 2009; Pellegrino et al., 2012), in our study only theta was different across conditions, but no other frequency bands. Notably, the slope of the relationship between theta and motor performance did not significantly differ for motor re-evaluation and motor re-programming, suggesting that theta (but not other frequency bands) is functionally relevant when the cue-movement associative rule is non-routine (Figure 3).

Second, our source analysis revealed that, although theta is a signature generated over a large network covering multiple fronto-central-parietal regions, the activity mostly relevant to motor performance was generated in L dPMC. Previous studies demonstrated that theta can be generated locally, within the sensori-motor regions during the late stages of motor learning (Perfetti et al., 2011b), in the fronto-parietal cortex for motor planning and on-line motor adjustments (Perfetti et al., 2011a) and as signature of brain plasticity in both physiological and pathological conditions (Kirov et al., 2009; Hung et al., 2013; Assenza et al., 2015; Pellegrino et al., 2016c; Tombini et al., 2012). As our task was explicitly designed to engage L dPMC, which is well known for its role in motor control, we could infer that this specific region generates theta waves to perform both motor re-evaluation and motor reprogramming. In a more general perspective, and in comparison with the view of theta activity being a widespread and non-specific marker of cognitive load, we support the idea that theta oscillations can be generated locally and their functional role is closely dependent on the specific role of their generator. That said, it is not surprising that other cortical areas showed an increase of theta, as L dPMC is a key area in a large dorsal network subserving motor control and motor integration (Ward et al., 2010; Hartwigsen et al., 2012; Moisa et al., 2012; Giambattistelli et al., 2014; Hartwigsen and Siebner, 2015). It is therefore likely that theta activity across these areas improves sensory-motor integration (Tombini et al., 2009; Cohen and Ridderinkhof, 2013; Figure 5). This interpretation is further corroborated by the evaluation of the relationship between the dynamic of L dPMC theta and motor performance *within* experimental conditions. Indeed, motor performance depended on both the amount and temporal dynamic of theta generation: the higher was theta, the shorter was the RT; the earlier theta reached its peak the quicker was the movement (Figure 5). As the relationship was similar for all three conditions -including the *Fully Congruent* trials where there was no violation of the associative rule- we might infer on a general positive values of theta oscillations in improving motor performance. In other words, this might suggest that increased theta does not only compensate an increased cognitive load (Laukka et al., 1995; Jensen and Tesche, 2002; Nigbur et al., 2011; Hsieh and Ranganath, 2014), but is instrumental to an accurate and quick motor act.

The studies performed by the group of Rushworth in healthy subjects as well as patients affected by stroke, had already demonstrated that L dPMC is part of a network involved in the so called “motor attention.” This network comprises two key nodes: the L dPMC, which is mainly devoted to selection of movements, and the left anterior inferior parietal lobule and the posterior superior parietal lobule which would be more involved in the preparation and the redirection of movements and movement intentions. For instance, stroke patients with left hemispheric lesions often show higher BOLD activation in the healthy PMC and the strongest motor impairment when this region is temporarily inactivated via TMS (Rushworth et al., 2003; Rushworth and Taylor, 2006).

Some limitations of this study should be underlined. First, a partial limitation is that EEG source analysis was not based (seeded) on individual MRI imaging. The tomographic reconstruction of EEG activity revealed, however, very broad maps, in keeping with the knowledge that motor system is organized over large cortical regions and networks (Raffin et al., 2015). Second, we cannot rule out that some theta activity localized over cortical surface was in fact generated in deeper regions, such as ACC and STN. We did not directly explore these regions as they are too deep to be properly sampled by our source imaging approach. Third, the analysis of theta activity time-dynamic was eventually restricted to the region most relevant to the task. However, future investigations should pay devote more attention to the time-dynamic of topographical maps.

In conclusion, our data confirm the notion that PMC is implicated in action re-evaluation and reprogramming and its signature in terms of brain oscillations is in theta band. This theta activity might be a good target for brain stimulation or other interventions to improve action selection in a damaged brain.

## Author Contributions

GP: experimental design, data acquisition, data analysis, data interpretation and manuscript preparation. LT: data analysis and manuscript revision. DH: experimental design, data interpretation and manuscript revision. KL: data acquisition. HS: experimental design, data acquisition, data interpretation, manuscript preparation and revision.

## Conflict of Interest Statement

The authors declare that the research was conducted in the absence of any commercial or financial relationships that could be construed as a potential conflict of interest.
